# Plants and low oxygen: from sensing and signalling to ecological adaptation and crop resilience

**DOI:** 10.1093/jxb/erag440

**Published:** 2026-10-30

**Authors:** Takeshi Fukao, Motoyuki Ashikari, Mikio Nakazono

**Affiliations:** Department of Bioscience and Biotechnology, Fukui Prefectural University, Fukui 910-1195, Japan; Bioscience and Biotechnology Center, Nagoya University, Nagoya 464-8601, Japan; Graduate School of Bioagricultural Sciences, Nagoya University, Nagoya 464-8601, Japan; UWA School of Agriculture and Environment, The University of Western Australia, WA 6009, Australia

**Keywords:** Anaerobic germination, flooding, hypoxia, oxygen sensing, redox signalling, submergence, waterlogging, wetland adaptation

**Low oxygen is a recurring feature of plant life, arising as both an environmental stress and a developmental condition. It is imposed by waterlogging, flooding, and submergence, but it also occurs within organs and tissues where oxygen demand exceeds supply. This Special Issue, Plants and low oxygen, brings together 14 reviews and one research article on how plants sense, signal, tolerate, avoid, and recover from oxygen limitation. Together, they show that low-oxygen biology now spans molecular sensing, redox and gasotransmitter signalling, systemic metabolism, anatomical adaptation, ecological diversity, and crop resilience (Box 1).**

Box 1. **A multi-scale view of plant low-oxygen biology and resilience**

Low-oxygen biology spans processes from molecular oxygen sensing to crop performance in flood-prone environments. The contributions to this Special Issue highlight several connected scales:

1. **Molecular:** oxygen sensing through the PCO/N-degron pathway, ERF-VII regulation, and redox and gasotransmitter signalling.
2. **Metabolic:** carbohydrate conservation, fermentation, antioxidant control, and metabolic exchange between hypoxic and normoxic tissues.
3. **Anatomical:** aerenchyma, adventitious and aerial roots, lenticels, radial oxygen loss barriers, leaf-surface wettability, and regulation of oxygen demand.
4. **Physiological:** nutrient acquisition, ion homeostasis, elemental redistribution, and recovery after reoxygenation.
5. **Ecological and agricultural:** adaptive trait combinations in wetland species and wild relatives, and their deployment in crop breeding for defined flooding regimes.

Together, these scales connect cellular responses to whole-plant survival, ecological adaptation, and the development of flood-resilient crops.

Oxygen is essential for plant metabolism as the terminal electron acceptor in mitochondrial respiration, a substrate for oxygen-dependent biochemical reactions, and a signal that shapes development and stress responses. Plant access to oxygen, however, varies strongly in space and time. Flooding restricts gas diffusion and is the most visible cause of plant hypoxia, but low-oxygen biology now extends beyond this classical stress framework. Earlier syntheses established that plants survive flooding through combinations of escape, quiescence, metabolic acclimation, and recovery, often involving ethylene, carbohydrate economy, aerenchyma, adventitious roots, and radial oxygen loss barriers (Bailey-Serres and Voesenek, 2008; Voesenek and Bailey-Serres, 2015). More recent work has sharpened this view by placing oxygen-dependent protein stability, hypoxia–reoxygenation stress, developmental hypoxic niches, and field-relevant crop resilience at the centre of the discussion (Loreti and Perata, 2020; León *et al*., 2021; Daniel and Hartman, 2024; Fagerstedt *et al.*, 2024). The field has therefore moved from cataloguing individual tolerance mechanisms towards understanding low-oxygen resilience as a multi-scale property of cells, organs, whole plants, ecosystems, and breeding systems.

## Low oxygen as stress, signal, and spatial gradient

Flooding remains the most familiar context for research on plant low-oxygen responses. When soil pores fill with water or aerial tissues are submerged, oxygen availability declines, constraining aerobic respiration. The consequences include reduced ATP production, altered ion homeostasis, carbon starvation, oxidative damage after reoxygenation, and impaired growth or survival. Yet the contributions to this issue place flooding-induced hypoxia within a broader biological framework: low oxygen is not only a damaging external stress but also an internal physiological condition and, in some contexts, a developmental signal.

Oxygen gradients occur naturally within plant bodies. Germinating seeds, meristems, vascular tissues, nodules, bulky organs, and growing leaves may contain hypoxic zones even under non-flooded conditions. Thus, a plant does not experience a single oxygen concentration; each organ and cell type experiences a particular combination of oxygen supply, respiratory demand, carbohydrate status, redox state, hormonal environment, and developmental programme. Manrique Gil *et al.* (2026) review recent advances in plant oxygen sensing and emphasize that the Plant Cysteine Oxidase (PCO)/N-degron pathway links oxygen availability to both environmental responses and development. Rodriguez-Cisneros *et al*. (2026) extend this view by framing flooding resilience as spatiotemporal signal integration, through which plants anticipate, acclimate to, and recover from flooding-induced hypoxia. Together, these reviews establish one of the main themes of the issue: oxygen availability is interpreted through context, not in isolation.

## Switching hypoxia responses on and off

The PCO/N-degron pathway is the molecular core of plant oxygen sensing. Under oxygen-replete conditions, PCOs promote the oxidation of N-terminal cysteine residues in group VII Ethylene Response Factors (ERF-VIIs), targeting these proteins for degradation. When oxygen availability is limited, ERF-VIIs become stabilized and activate hypoxia-responsive genes that support anaerobic metabolism and survival. Castellana *et al.* (2026) focus on how this response is switched on and off. Their review highlights that the hypoxic transcriptional response is not controlled by oxygen-dependent ERF-VII stability alone, but is fine-tuned by additional regulatory layers, including co-regulator complexes, Target of Rapamycin (TOR)-dependent energy signalling, calcium signalling, and transcriptional repressors that attenuate hypoxia-responsive gene expression.

This emphasis on switching off is especially important. Reoxygenation does not simply restore the pre-stress state. Although renewed oxygen availability should promote ERF-VII degradation, this process may be delayed or modified by reactive oxygen species (ROS) and other signals. The output of the hypoxia programme, therefore, depends on timing, intensity, tissue type, and the transition back to oxygenated conditions. Understanding the termination and resetting of hypoxia responses is likely to be as important as understanding their activation.

## Redox and gasotransmitter networks

Redox and gasotransmitter pathways add further regulatory depth. Nitric oxide (NO), ROS, and hydrogen sulfide (H_2_S) can be damaging at high concentrations, but they also act as information-rich signals that influence protein stability, enzyme activity, hormone responses, and mitochondrial function. van der Wijk *et al*. (2026) review phytoglobin 1 (PGB1) as a hub linking hypoxia acclimation, NO homeostasis, redox balance, and development. PGB1 can influence ERF-VII stability through NO scavenging, support alternative energy generation through the PGB1–NO cycle, mitigate oxidative stress, and help maintain auxin transport. Telara *et al*. (2026) examine H_2_S and its derivatives, with particular emphasis on protein persulfidation, and discuss how H_2_S-derived signals interact with NO and ROS during low-oxygen responses.

These reviews reinforce a key conceptual shift. Low-oxygen signalling is not a linear chain from oxygen concentration to gene expression. Rather, it is a network in which oxygen sensing, redox state, energy status, hormone signalling, and gasotransmitters continuously modify one another. This networked view helps explain why similar oxygen treatments can produce different outcomes depending on the plant developmental stage, tissue identity, nutrient status, and the severity or duration of flooding.

## Oxygen supply, oxygen demand, and whole-plant function

Molecular signalling operates within physical constraints. Because oxygen moves largely by diffusion, tissue porosity, organ size, respiratory demand, and the spatial arrangement of hypoxic and aerated tissues influence whether oxygen limitation becomes damaging. Jiménez and Pedersen (2026) provide a conceptual framework in which maximum root length under flooding depends not only on improved oxygen diffusion through aerenchyma but also on tissue-specific oxygen consumption. Their analysis emphasizes that lowering respiratory demand can be as effective as increasing porosity, and that the benefits of additional porosity diminish once gas-phase diffusion becomes efficient.

Several contributions place this principle in broader settings. Striker *et al*. (2026) review maize responses to waterlogging, integrating aerenchyma formation, adventitious roots, ethylene–auxin interactions, radial oxygen loss barriers, shoot photosynthesis, and senescence. Inoue (2026) examines mangrove aeration systems, in which aerial roots, lenticels, and connected lacunae withstand repeated tidal flooding but do not eliminate root hypoxia during prolonged inundation. Hashimoto and Itoh (2026) add a complementary surface perspective by reviewing the roles and regulation of leaf hydrophobicity, a trait that influences water retention, gas exchange, pathogen interactions, and plant–water contact during wet conditions. Together, these papers show that plant oxygen status emerges from anatomy, surface properties, metabolism, and environmental dynamics.

## Systemic metabolism, ions, and recovery

A plant experiencing waterlogging often contains both hypoxic and normoxic tissues. Herfurth *et al.* (2026) introduce ‘metabolic snorkeling’ to describe metabolic cooperation between tissues with different oxygen availabilities. Hypoxia-related metabolites produced in roots can be transported to shoots, where they may be recycled, while carbohydrate supply from aerated tissues can support metabolism in hypoxic tissues. This concept shifts attention from local acclimation to whole-plant metabolic integration.

Recovery after oxygen deprivation is another key stage. Sarkar *et al*. (2026) use time-series elemental imaging in *Arabidopsis* cation/H^+^ exchanger mutants to examine elemental redistribution during recovery from anoxia. This research article advances the field by linking post-anoxia performance to the spatial distribution of nutrients and metals. Yun and Shabala (2026) broaden this issue by arguing that waterlogging tolerance research has focused heavily on oxygen supply traits while underemphasizing nutrient acquisition, ion homeostasis, and elemental toxicities caused by changes in soil redox potential. Their comparison of wetland and dryland species suggests that flood-adapted plants may possess higher copy numbers of genes involved in nutrient acquisition and tolerance to elemental toxicity. This perspective is timely because flooding is not just a form of oxygen stress; it is also a nutrient, ion, redox, and recovery challenge.

## Ecological diversity and crop resilience

Natural diversity provides a critical counterweight to model-based and crop-centred research. Mustroph and Mandáková (2026) synthesize adaptation in wild Brassicaceae, especially water-associated lineages such as Cardamineae. Their review shows that flood adaptation can involve aerenchyma, adventitious roots, heterophylly, growth modulation, and context-dependent effects of genome duplication. Importantly, natural habitats often combine flooding with salinity, low temperature, mechanical disturbance, or drought transitions. Such complexity makes wild relatives valuable not only as sources of traits but also as models for understanding how combinations of traits function in fluctuating environments.

A major applied goal is to translate mechanistic understanding into crops that can establish, survive, recover, and yield under variable flooding regimes. Flooding may occur during germination, seedling establishment, vegetative growth, or reproduction, and it may take the form of transient submergence, stagnant flooding, deepwater inundation, or waterlogging with nutrient stress. Rice is the clearest example of successful translation. Lamsal *et al.* (2026) examine two stage-specific rice traits: anaerobic germination and vegetative-stage submergence tolerance. The contrast is instructive: anaerobic germination requires an escape-like programme of rapid coleoptile elongation, supported by starch mobilization and sugar signalling, whereas *SUBMERGENCE1A* (*SUB1A*)-mediated submergence tolerance at later stages favours quiescence, carbohydrate conservation, and recovery. Hussain *et al.* (2026) extend this view through a 5D breeding framework—Demand, Discovery, Design, Development, and Deployment—arguing that single-gene introgression is insufficient for the diversity of flood-prone rice environments and must be integrated with genomic selection, high-throughput phenotyping, target-environment definition, and deployment feedback.

## Designing low-oxygen resilience

The articles in this issue point to a design-oriented phase in low-oxygen research on plants. The starting point should be a clearly defined flooding scenario. Is the target anaerobic germination, transient complete submergence, soil waterlogging, stagnant flooding, deepwater escape, or tidal inundation? At which developmental stage does stress occur, and what co-stresses accompany it? Different answers require different trait combinations. Escape growth can be valuable in deepwater environments but costly during complete submergence if carbohydrate reserves are depleted. Quiescence can improve survival during prolonged submergence but may delay recovery after short floods. Enhanced aerenchyma and radial oxygen loss barriers can improve internal aeration but may interact with nutrient uptake, root strength, and microbial processes. Reduced oxygen consumption can extend aerated root length but may constrain growth if not carefully tuned. Nutrient-acquisition traits may improve performance in waterlogged soils but could involve trade-offs under drained conditions.

Several questions now stand out. How do plants integrate oxygen, energy, redox, nutrients, and hormone signals across tissues? How are hypoxia responses terminated during reoxygenation? Which responses observed under laboratory hypoxia are causally involved in field tolerance, and which are secondary consequences? How can developmental hypoxic niches be separated from stress-induced hypoxia, and how do natural habitats shape the evolution of low-oxygen responses? Answering these questions will require spatial and temporal tools that match the complexity of low-oxygen biology: oxygen microsensors, tissue-specific reporters, time-resolved imaging, spatial omics, metabolite flux analysis, elemental imaging, and phenotyping under realistic flooding conditions.

The message emerging from this Special Issue is that low-oxygen biology is no longer a single-stress field. It is a framework for understanding how plants manage gas diffusion, energy limitation, redox balance, nutrient stress, developmental programmes, and ecological variability. Future progress will depend on connecting molecular switches to whole-plant design principles and on testing those principles in the environments where crops and wild plants actually encounter low oxygen.
